# Evaluating patient harm minimization during the COVID-19-driven reduction in benign gynecological care: a nationwide claims-based longitudinal study in the Netherlands

**DOI:** 10.1371/journal.pone.0345619

**Published:** 2026-04-01

**Authors:** Eva L.M. Velthuijs, Ismail Ismail, Robert A. de Leeuw, Wouter J.K. Hehenkamp, Xander H.E. Koolman

**Affiliations:** 1 Department of Obstetrics and Gynecology, Amsterdam UMC, Location VU/AMC, Amsterdam, The Netherlands; 2 Department of Health Economics, Vrije Universiteit, Amsterdam, The Netherlands; Medical Park Minatomirai, JAPAN

## Abstract

**Objective:**

To evaluate whether the COVID-19 pandemic influenced treatment modality, care intensity, and care setting in benign gynecological care in the Netherlands.

**Design:**

Retrospective longitudinal cohort study evaluating the effects of the COVID-19 pandemic.

**Setting:**

Nationwide healthcare delivery was analyzed across four benign gynecological pathways from 2017 to 2022 using Vektis and Dutch Hospital Data (DHD), accessed via Statistics Netherlands (CBS).

**Participants:**

The study focused on four benign gynecological pathways, classified using Dutch Diagnosis Treatment Combinations (DTCs): menstrual disorders (G11), uterine fibroids (G15), prolapse (G25), and first-trimester pregnancy complications (Z12). All patients with a new DTC in the first half of each year were included. Exclusion criteria were patients under 18 years, and only in the menstrual disorder pathway, patients over 51 years of age.

**Intervention or exposure:**

Cohorts from the initial pandemic year (2020) were compared to pre-pandemic years (2017–2019), the late pandemic year (2021), and the post-pandemic year (2022).

**Main outcomes and measures:**

The primary outcome was the change in treatment mix within the care pathways. Secondary outcomes included regional variation, hospital-type distribution and care intensity.

**Results:**

In 2020, all care pathways showed reduced unique patient volumes. Treatment mix did change during the COVID-19 pandemic, but not uniformly across the four pathways toward more less-invasive options. We found no regional differences in treatment mixes. Patient age, number of care activities and medication use remained stable. Telephone follow-up consultations increased markedly in 2020. No deviation from the trend was observed in respect of hospital type providing care.

**Conclusions and relevance:**

A sharp reduction in benign gynecological care did not result in strategies to minimize patient harm like prioritization or substitution of care, as observed with our available claims data. Our findings indicate a lack of flexibility in health care provision to adjust to rapidly changing demands. Data limitations underscore the need for complementary research using other data sources to assess patient harm.

## Introduction

The COVID-19 pandemic disrupted routine healthcare delivery abruptly and reduced access to elective surgeries. This created a unique natural experiment to assess the necessity and effectiveness of standard care. With intensive care units overwhelmed by critically ill patients and operating room capacity repurposed for COVID-related services, hospitals worldwide were forced to postpone or cancel elective procedures [1–6]. Across countries, elective care volumes dropped significantly during the pandemic, although the extent and duration of disruption varied. In Germany, a multicenter study reported a 25.6% decrease in elective benign surgeries, particularly for procedures such as thyroidectomy and hernia repair. Emergency surgeries declined by 18.8% [7]. In the United Kingdom, delays in joint replacement procedures have persisted well beyond the acute phase of the pandemic [8]. In the Netherlands, elective surgery volumes declined sharply during pandemic peaks, while acute care remained relatively stable [9]. These patterns suggest that benign elective care, often considered deferrable, was particularly affected as systems prioritized life-threatening and urgent conditions.

Although these studies have documented substantial declines in elective care, particularly in surgical specialisms, it remains unclear how healthcare systems responded. Some may have substituted invasive surgical procedures with less intensive alternatives, such as outpatient interventions or pharmacologic management. Others may have deferred care without replacement [1,10–14]. Few analyses have examined whether hospitals, under resource constraints, adapted their care delivery by prioritizing certain patients or interventions [10,14–16]. Consequently, it is unclear to what extent the observed reductions reflect temporary service delays or more deliberate changes in care intensity and patient selection. Addressing this gap is crucial for understanding how healthcare systems operate under pressure. These insights are increasingly relevant given the ongoing financial constraints, staff shortages, the need for more sustainable models of care, and the necessity for pandemic preparedness to ensure resilience in future health crises [17–20].

Benign gynecological care is especially relevant in this context. It includes multiple care pathways where surgical procedures have well-established non-surgical alternatives, such as hormonal treatments, intrauterine devices or minimally invasive outpatient interventions. During periods of constrained access to operating theatres, such alternatives may have been used more frequently to manage symptoms or bridge care gaps. This makes benign gynecology an informative domain for studying how care systems triaged patients and whether substitution occurred under pressure. In previous work, we observed that in the Netherlands, both surgical and non-surgical care for benign gynecological conditions decreased markedly during the first wave of the pandemic [21]. Contrary to the hypothesis that surgical procedures might be replaced by non-surgical alternatives, we found little evidence of such substitution. Instead, care appeared to have been scaled down across the board, raising important questions about how care delivery was adapted under pressure.

While these earlier findings highlighted overall reductions in volume, the current study takes a more granular approach and investigates how care delivery was reshaped to meet acute shortages of health care in order to minimize the consequences for patients. We focus on the combination of treatment modality (surgical vs non-surgical), care intensity and care setting (hospital type and region). We hypothesize that under resource constraints, hospitals may have adjusted the mix of health care provided and the selection of patients treated by prioritizing certain patient groups (triage), decentralizing care (hospital type or region) or substituting surgical for non-surgical treatment (particularly through increased use of pharmacologic alternatives).

Therefore, this study adds to existing research by focusing on the efforts made to minimize patient harm, using national-level claims data from 2017 through 2022. We explore this using changes in treatment delivery. This analysis offers insight into how the Dutch health system adapted benign gynecological care under pandemic conditions and informs the broader transition toward more resilient, appropriate, and sustainable care models in gynecology and beyond.

## Methods

### Study design and setting

We conducted a retrospective, longitudinal study to evaluate the impact of the COVID-19 pandemic on treatment patterns within four benign gynecological care pathways in the Netherlands. The first confirmed COVID-19 case was reported on February 27, 2020 [22]. A nationwide lockdown followed on March 15, 2020, including the closure of schools, hospitality venues, and non-essential services, as well as the suspension of non-urgent medical care. After a period of relative calm in the summer, a second partial lockdown was introduced on October 14, 2020, and intensified into a stricter lockdown on December 14, 2020, lasting into early 2021. A third lockdown began in mid-December 2021 in response to the Omicron variant and continued until January 25, 2022 [23]. Throughout these periods, hospitals faced high Intensive Care Unit (ICU) occupancy, especially during peaks in April 2020 and January 2021 [24]. Vaccination started January 6, 2021, gradually easing the pressure on intensive care units, but care backlogs and staff shortages persisted well into 2022 [24,25]. Only from spring 2022 onward did hospital operations begin to normalize, allowing more consistent access to elective and outpatient care.

We compared care delivered during the first pandemic year (2020), the later pandemic period (2021), and the post-pandemic year (2022) with care provided in the pre-pandemic period (2017–2019). Each care pathway reflects a defined clinical trajectory, encompassing all related consultations, procedures, and treatments. The post-pandemic period was limited to 2022 due to the unavailability of data beyond that year at the time of analysis.

The pathways were identified using Dutch Diagnosis Treatment Combinations (DTCs) and included menstrual disorders (G11), uterine fibroids (G15), pelvic organ prolapse (G25), and first-trimester pregnancy complications (Z12). Dutch DTCs are a classification system used within the Dutch healthcare system to define patient care pathways based on both diagnoses and treatments. Each DTC reflects a clinically relevant care trajectory that groups together related consultations, procedures, and treatments for specific conditions. This system is used to standardize and structure healthcare data, enabling detailed tracking and analysis of care provided in hospital healthcare settings. Pathways were selected by the authors based on the availability of similarly effective non-surgical alternatives to standard high volume surgical procedures [26–30], allowing analysis of possible changes in treatment mix across different types of care. For example, in heavy menstrual bleeding, hysterectomy is often performed, but less invasive options such as endometrial ablation or hormonal intrauterine devices (IUDs) are available and do not require operating theatre resources. For uterine fibroids, hysterectomy is commonly used, while uterine artery embolization offers a similarly effective non-surgical alternative. Pelvic organ prolapse is frequently managed surgically with vaginal wall repair, although conservative treatment with a pessary can be initiated in an outpatient setting. Similarly, first-trimester pregnancy complications such as miscarriage are often treated with surgical uterine evacuation, but can also be managed expectantly or with medication such as misoprostol.

Two pathways were excluded from this study (infertility treatment (F11) and endometriosis (G17), as opposed to our earlier work [21], as these involved fewer surgical interventions and had limited potential for substitution with non-surgical alternatives. Excluding these pathways allowed for a more coherent narrative focused on conditions where substitution between surgical and non-surgical care was meaningful and measurable.

To assess whether changes in treatment mix varied geographically, we examined regional variation in the burden of COVID-19 and their potential influence on gynecological care delivery. When looking at the distribution of COVID-19 hospitalizations per province in the Netherlands during the period March–June 2020, we see that the southern provinces of Noord-Brabant and Limburg were most affected, while northern provinces such as Friesland, Drenthe, and Groningen reported substantially lower hospitalization rates [31]. The impact of the pandemic was also shaped by the underlying distribution of ICU infrastructure: most intensive care units are concentrated in the more densely populated western provinces, particularly the Randstad region (e.g., Noord-Holland, Zuid-Holland, and Utrecht), whereas the northern provinces have fewer participating ICUs [32]. By linking treatment patterns to patients’ province of residence, we aimed to identify whether care delivery adapted differently across the country in response to these pressures.

### Ethics statement

This study involves human participants but was not approved by an Ethics Committee or Institutional Review Board. We used Statistics Netherlands (Centraal Bureau voor de Statistiek, CBS) microdata for our research. As part of the data application process, CBS reviews whether the study complies with legal and ethical guidelines before granting access. All insurance records were fully anonymized prior to researcher access and analyzed within the secured CBS environment. Since the study is retrospective and based on anonymized, non-identifiable data, it does not fall under the scope of the Medical Research Involving Human Subjects Act (WMO) and the requirement for informed consent was waived. Further details on data access conditions and restrictions are provided in the Data Availability Statement.

### Data collection

We used routinely collected administrative and clinical data available through CBS which integrates national claims data from Vektis and hospital care data from Dutch Hospital Data (DHD). Vektis compiles health insurance claims data, while DHD provides detailed hospital activity records derived from electronic health records, including diagnostic, procedural, and admission data. These sources were linked at the patient level using anonymized identifiers within the CBS secure research environment. We accessed the CBS data between 1^st^ of July 2024 and 1^st^ of March 2025. We had no access to information that could identify individual participants during or after data collection and there were no external organizations involved in the collection or provision of the data.

Care pathways were defined using Dutch DTC codes and associated care activity codes. Care activity codes register specific medical actions, such as performing an ultrasound, placing an IUD, conducting an outpatient consultation, or conducting a surgical procedure. These codes allow for detailed differentiation between types and settings of care provided within each DTC. Medication use was incorporated by linking relevant Anatomic-Therapeutic-Chemical-4 codes (ATC-4) codes to individual records. Important to notice is that ATC-4 codes give no specification in names of medication included in a group and also do not give a date of use. Data were stratified by region, categorized as North, Middle, or South based on Dutch provinces. We also analyzed variation by hospital type, distinguishing between academic, top-clinical, general hospitals, and independent treatment centers (ITCs), as predefined by CBS. Academic hospitals are university-affiliated tertiary care centers that provide complex and highly specialized care alongside teaching and research. Top-clinical hospitals are large general hospitals with additional functions such as specialized services and teaching responsibilities, but without a university affiliation. General hospitals provide a broad range of standard secondary care services without a formal teaching or tertiary function. ITCs are privately operated facilities that typically focus on high-efficiency, low-complexity elective care, often in outpatient settings and without emergency services. Stratifying analyses by hospital type allowed us to examine whether patterns of care delivery differed across hospital categories and whether any observed shifts were sustained over time, providing insight into how healthcare resources were utilized efficiently.

### Population

We included all patients with a new DTC opened between January and June in each calendar year from 2017 through 2022. Patients under the age of 18 were excluded from all pathways. For the menstrual disorders pathway, patients older than 51 were excluded to minimize the inclusion of postmenopausal cases (the DTC of cycle abnormalities consists of patients with heavy menstrual bleeding and postmenopausal bleeding). Each patient was assigned to the year in which the DTC episode began, regardless of when individual care activities occurred during the follow-up period.

### Dataset processing

Data processing and linkage were performed using RStudio within the CBS secure environment. Patient-level records were constructed by merging datasets using anonymized identifiers, resulting in a unified dataset with variables including DTC code, care activity type and date, start and end dates of DTCs, age, hospital type, region, and medication codes. Inclusion and exclusion criteria were applied after the dataset was constructed. The final dataset comprised all relevant DTC episodes and care activities for the four gynecological pathways over the six-year study period.

### Outcome measures

The primary outcomes were the trends in treatment mix within the different care pathways, referring to the relative use of surgical procedures, outpatient interventions, and pharmacological treatments. These patterns reflect how care delivery may have shifted under pandemic-related constraints. Secondary outcomes included regional variation in treatment mix, distribution of patients per hospital category (Academic hospital, Top clinical hospital, Peripheral hospital, ITC), and patient selection. Because clinical severity is not directly observable in our dataset, we used proxies such as mean age and average number of care activities per patient to assess potential shifts in patient selection. If these remained stable, it might have suggested that triage based on urgency or complexity did not occur systematically. Conversely, any significant changes might have reflected prioritization under resource constraints.

### Statistical analysis

Descriptive statistics were used to compare care delivery across periods. Results are presented as proportions or means, as appropriate. Statistical comparisons between proportions were conducted using chi-square tests, and differences in means were assessed using independent-samples t-tests. Normality of continuous variables was assessed using the Shapiro-Wilk test and visual inspection of histograms and Q-Q plots. For non-normally distributed continuous variables, non-parametric alternatives such as the Mann-Whitney U test were used. Statistical significance was defined as a two-sided p-value < 0.05. No imputation was performed for missing data. All analyses were conducted in RStudio (version 4.2.1).

## Results

A total of 323,946 patient records across the four care pathways were included for the years 2017–2022. Table 1 shows baseline characteristics for each pathway during this period.

**Table 1:** Characteristics of care pathways per year.

	2017	2018	2019	2020	2021	2022
**Heavy menstrual bleeding**						
Unique patients (N)	33298	29994	27441	20341	23713	20310
Age (mean) 95% Confidence Interval	37.43 37.33–37.52	37.19 37.08–37.29	36.90 36.79–37.01	36.29 36.16–36.43	35.62 35.49–35.75	35.36 35.22–35.50
Number of care activities (mean)* 95% Confidence Interval	1.83 1.81–1.84	1.76 1.75–1.77	1.66 1.65–1.68	1.65 1.64–1.67	1.50 1.49–1.51	1.37 1.36–1.38
**Uterine fibroids**						
Unique patients (N)	6170	4944	4320	3120	3337	3118
Age (mean) 95% Confidence Interval	47.70 47.46–47.95	48.11 47.83–48.38	48.23 47.92–48.54	48.76 48.39–49.14	48.35 47.99–48.71	49.03 48.64–49.43
Number of care activities (mean)* 95% Confidence Interval	2.61 2.55–2.67	2.43 2.37–2.48	2.29 2.23–2.34	2.26 2.21–2.32	2.02 1.98–2.06	1.69 1.66–1.72
**Prolapse**						
Unique patients (N)	19480	17739	16802	11292	13067	13873
Age (mean) 95% Confidence Interval	60.32 60.11–60.54	60.75 60.53–60.97	61.40 61.17–61.62	61.75 61.47–62.03	62.25 61.99–62.51	63.36 63.12–63.61
Number of care activities (mean)* 95% Confidence Interval	2.76 2.72–2.79	2.55 2.52–2.58	2.40 2.37–2.43	2.32 2.29–2.36	2.02 2.00–2.04	1.67 1.66–1.68
**First trimester pregnancy problems**						
Unique patients (N)	15844	14298	12444	10483	10112	8716
Age (mean) 95% Confidence Interval	32.14 32.05–32.23	32.05 31.95–32.14	32.09 31.99−32.20	31.89 31.78–32.00	31.91 31.80–32.02	31.65 31.53–31.77
Number of care activities (mean)* 95% Confidence Interval	1.46 1.45–1.48	1.43 1.41–1.44	1.40 1.39–1.42	1.36 1.34–1.37	1.30 1.29–1.31	1.21 1.1–1.22

* = average number of care activities per patient within the group Grey markdown = Initial Covid-19 period

The care pathway for heavy menstrual bleeding had the largest volume of patients, while uterine fibroids had the smallest. In 2020 the volumes were lowest in all care pathways except first-trimester pregnancy problems. We see no deviation in 2020 from the pre-existing trend of either increasing or decreasing mean age over the years. Besides, the mean number of care activities per patient does not deviate from the trends in any of the pathways in 2020.

Table 2 shows the treatment mix of surgical and, if relevant, non-surgical treatment options within the care pathways. Shown are proportions of the treatment types on the total of all the included treatments combined within a pathway.

**Table 2:** Treatment mix per care pathway per year.

	2017	2018	2019	2020	2021	2022
**Heavy menstrual bleeding**						
Etonorgestrel contraceptive implant insertion	0.33	0.63*	0.49	0.31	0.39	0.67
Intrauterine device insertion	7.73	8.12	9.84*	9.76	11.03	10.37
Diagnostic hysteroscopy	4.31	3.78	4.14	5.10*	3.94*	4.10
Therapeutic hysteroscopy	21.34	20.54	21.51	22.39	21.79	20.83
Hysteroscopic myomectomy (TCRM) type a	13.04	12.29	12.39	12.86	14.99*	16.14
Hysteroscopic myomectomy (TCRM) type b	33.35	36.06*	35.71	34.01	34.33	36.29
Diagnostic laparoscopy	1.47	1.57	1.58	1.53	1.76	2.02
Total laparoscopic hysterectomy	11.60	11.37	10.21*	10.14	8.83*	6.88*
Vaginal hysterectomy	5.41	4.40*	2.97*	2.96	2.36	2.02
Abdominal hysterectomy	1.43	1.22	1.16	0.94	0.57	0.67
**Uterine fibroids**						
Intrauterine device insertion	1.82	1.84	1.35	2.29	1.99	2.15
Diagnostic hysteroscopy	3.12	2.57	2.76	2.76	2.79	2.46
Therapeutic hysteroscopy	8.14	8.35	9.68	11.51	10.04	9.02
Uterine artery embolization	0.36	0.29	0.45	0.47	0.24	0.20
Hysteroscopic myomectomy (TCRM) type a	12.29	10.83	11.54	11.74	14.18	16.70
Hysteroscopic myomectomy (TCRM) type b	19.53	20.15	22.23	21.75	23.82	25.31
Myomectomy	7.75	8.01	6.13*	6.30	7.97	7.07
Diagnostic laparoscopy	0.95	1.75*	1.13	0.87	1.20	0.82
Total laparoscopic hysterectomy	22.96	23.01	21.78	21.51	17.53*	15.37
Vaginal hysterectomy	2.53	1.41*	2.19	1.34	1.27	1.23
Abdominal hysterectomy	20.55	21.80	20.77	19.46	18.96	19.67
**Pelvic organ prolapse**						
Total laparoscopic hysterectomy	0.58	0.54	0.47	0.55	0.33	0.31
Vaginal hysterectomy	1.82	1.64	1.29	1.65	1.44	0.91
Anterior&Posterior (A&P) colporrhaphy	32.24	31.36	30.87	34.17	34.26	34.42
A&P colporrhaphy with cervical amputation	6.76	6.83	7.86*	7.65	9.24*	9.97
A&P colporrhaphy with vaginal hysterectomy	11.06	11.60	8.83*	7.71	6.98	5.67*
A/P colporrhaphy with mesh placement	1.18	0.93	0.65	0.78	0.43	0.70
A/P colporrhaphy with double mesh placement	0.64	0.37*	0.22	0.06	0.26	0.35
Mid-urethral sling placement (TVT/TOT) placement	16.34	17.17	17.11	16.01	14.15	14.76
TVT/TOT + A/P colporrhaphy	1.67	1.70	1.34	0.81*	0.98	1.19
Enterocele repair abdominal/vaginal	0.78	0.76	0.93	0.84	0.92	1.01
Vaginal vault suspension	0.67	0.53	0.26*	0.45	0.26	0.31
Sacro colpopexy	1.67	1.63	2.22	1.98	1.90	1.61
Sacro colpopexy + A/P colporrhaphy	20.87	21.87	24.16*	24.05	25.97	27.04
Sacro colpopexy + A&P colporrhaphy	1.14	0.92	1.18	0.71*	1.15	0.24*
Laparoscopic Sacro colpopexy	2.56	2.15	2.60*	2.56	1.74	1.50
**First trimester pregnancy complications**						
Hysteroscopy	2.12	2.99*	7.19*	9.87*	12.48*	11.61
Uterine curettage	9.86	9.04	8.38	7.50	10.24*	11.36
Surgical abortion/evacuation of retrained products of conception	71.85	71.90	66.43*	62.40*	56.30*	53.96
Laparoscopic surgery ectopic pregnancy	15.34	15.25	17.12*	19.21*	19.97	22.31
Open surgery ectopic pregnancy	0.82	0.81	0.88	1.03	1.02	0.76

Proportions of individual procedures within each care pathway are shown per year, summing to 100% within each pathway.

*P = < 0.05 compared to previous year.

When examining the treatment patterns within the four different care pathways, it becomes apparent that in the pathways heavy menstrual bleeding and uterine fibroids all types of surgical treatment options either do not deviate from existing decreasing trends or are below expected trend levels in 2020. Looking at the non-surgical treatment options, we see a slight increase in proportion in 2020 for both types of hysteroscopy and insertion of IUDs in the heavy menstrual bleeding pathway. In the uterine fibroids pathway, we see an increase in the insertion of IUDs, therapeutic hysteroscopies and uterine artery embolization in 2020. In the years 2021 and 2022 we see an increase in both pathways in the use of hysteroscopic myomectomies and in the heavy menstrual bleeding pathway of diagnostic laparoscopies as well.

In the prolapse pathway we only looked at surgical treatment options. We can see a shift in treatment mix towards more Anterior&Posterior (A&P) colporrhaphy and vaginal vault suspensions in 2020, while the combined A&P colporrhaphy procedures, mid-urethral sling (TVT/TOT) placements and sacro colpopexies remained stable or decreased in proportion. In the following years, the proportions of enterocele repair, sacro colpopexy in combination with A&P colporrhaphy and the A&P colporrhaphy in combination with cervical amputation rose in proportion.

In the first trimester pregnancy complications pathway an increase was seen in the proportion of hysteroscopies and ectopic pregnancy surgeries in 2020. In the years 2021 and 2022 the proportion of uterine curettage increased again. A decreasing trend is seen over the years of surgical abortion.

Additionally, we looked at changes in treatment mix within regions across the Netherlands during the study period (see S1 Tables A – D in S1 File). All three regions in our pathways show roughly the same patterns in treatment use across the years, however there are some differences between the regions within the pathways in 2020. In the pathway heavy menstrual bleeding, we see proportionally more diagnostic hysteroscopies in regions middle and south, while we see more therapeutic hysteroscopies in north, combined with fewer diagnostic laparoscopies. In the uterine fibroids pathway, more diagnostic hysteroscopies and myomectomies are seen in region north. In region south we see more therapeutic hysteroscopies in 2020. In region middle, the proportion of hysteroscopic myomectomies type b is increased. In the prolapse pathway, the proportion of vaginal hysterectomies was lower in region north. Region middle showed a higher proportion in total laparoscopic hysterectomies and region south had higher proportion of vaginal vault suspension, while showing lower proportion of A&P colporrhaphy. The pathway first trimester pregnancy complications only showed a differentiation in the proportion of open surgery for ectopic pregnancy, which was higher in the middle region.

**Fig 1:**
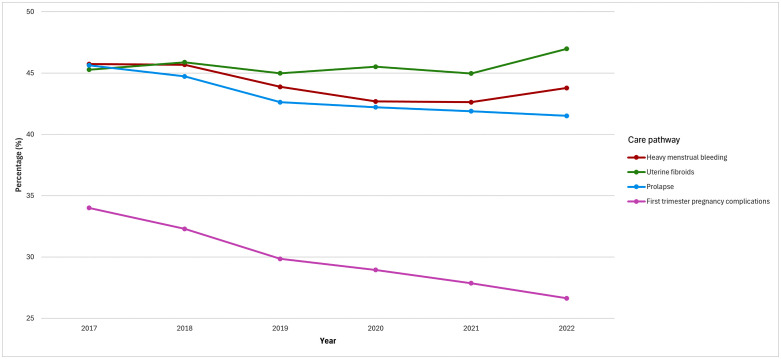
Percentage of medication use over all patients in a care pathway per year.

The proportion of medication use per year does not differ significantly from pre-pandemic levels during the COVID-19 pandemic (Fig 1). A small increase in use is observed in 2020 for the uterine fibroids pathway compared to the previous year (2019) and the following year (2021). However, the proportion is still in line with the pre-existing trend.

**Fig 2:**
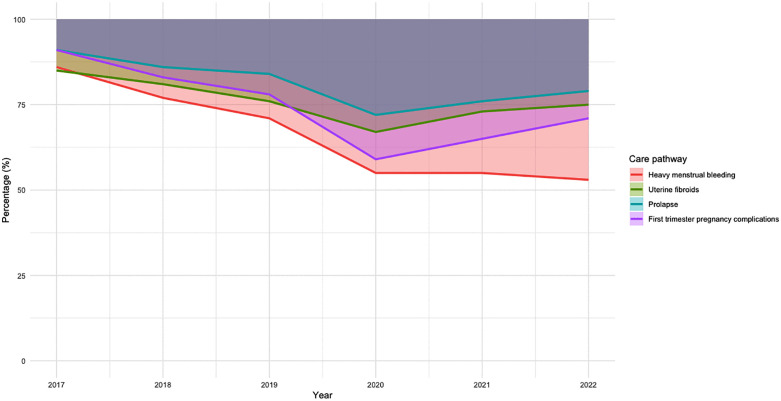
Proportion of telephone and physical follow-up consultations per year across all four care pathways (2017–2022). The colored area represents the percentage of telephone consultations, and the area below the line represents the percentage of physical consultations.

We see a trend towards more telephone follow-up consultations instead of physical follow-ups over the years (Fig 2). A *significant* deviation in this shift is seen in the year 2020 in all four pathways. Most increase is seen in the first trimester pregnancy complications pathway (19%) and least in the uterine fibroids pathway (9%) compared to proportions of 2019. This means that telephone consultations became much more prevalent after COVID and that this trend persisted for heavy menstrual bleeding.

**Fig 3:**
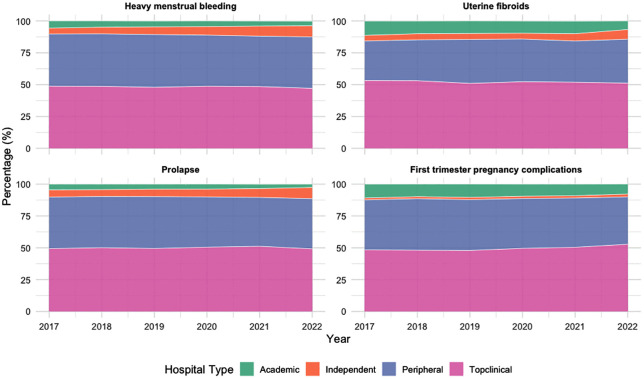
Distribution of hospital types providing care, expressed as proportion of total care per year across four benign gynecological pathways (2017–2022).

When looking at the types of hospitals where patients received care, we see that most care is delivered in both top clinical hospitals and peripheral hospitals in all four care pathways (Fig 3). Besides, we see a slight increase in proportion over the years in all four pathways toward care delivery in ITC’s, compensating for a decrease in care delivery in academic hospitals. No significant changes in proportions of hospital type were observed in any of the four care pathways within the study period.

## Discussion

This nationwide study investigates how benign gynecological care delivery in the Netherlands was reshaped during the COVID-19 pandemic to mitigate patient harm under conditions of acute resource scarcity. We examined how the health system adjusted treatment modalities, care intensity and care settings across four clinical pathways. Treatment mix changed during the COVID-19 pandemic, but not uniformly across the four pathways or years. While some shifts toward less invasive or outpatient alternatives were visible, particularly in the management of heavy menstrual bleeding and uterine fibroids, most surgical procedures followed existing trends or declined. In prolapse care, we observed internal shifts within surgical options. Overall, medication use did not significantly differ from pre-pandemic levels. Telephone consultations increased sharply in 2020, particularly for first trimester pregnancy complications (19%) and this shift persisted for heavy menstrual bleeding. We found no regional differences in treatment mixes. Patterns of care delivery across hospital types remained stable, with most care provided in top-clinical and peripheral hospitals. A modest increase in care delivery within ITCs was seen across pathways, compensating for a slight decrease in care within academic hospitals. We found that while overall care volumes declined, patterns of care delivery were largely stable. These findings suggest that system adaptations were limited and mostly aimed at maintaining continuity rather than transforming care delivery on a broader scale.

Across all pathways, patient age and care intensity (mean number of care activities) remained stable, indicating that hospitals continued to serve a similar patient population, without shifting toward more urgent or complex cases. This stability is important, as it suggests that any observed shifts in treatment delivery reflect changes in system-level response rather than shifts in our patient population itself [33]. Our findings align with previous findings that indicated care avoidance and deferral rather than redistribution during the pandemic [16,18,34,35].

Our analysis of treatment mixes in the different pathways show that only limited shifts occurred during the initial COVID-19 period. While guidelines at the time encouraged the use of less resource-intensive treatments when possible [11,36,37], our findings suggest that such substitutions were not widely implemented. This may reflect the reality that many hospitals redirected capacity almost entirely to acute and COVID-related care, leaving little room for proactive restructuring of benign services [38]. Moreover, Dutch clinical guidelines for benign gynaecology permit considerable clinical discretion, allowing physicians, within reason, to deviate from recommended surgical pathways based on patient-specific factors, preferences, and shared decision-making. Compared to more centrally regulated healthcare systems, the Netherlands affords clinicians relatively high professional autonomy in treatment decisions, which may have further enabled selective rather than systematic substitution during capacity constraints. Even in the post-acute phase of the pandemic, the reorganization of benign gynecological care appears to have progressed only gradually. This limited restructuring likely reflects persistent uncertainty regarding the consequences of deferred or modified care, including potential impacts on waiting times, quality of life, and long-term outcomes. At that time, evidence was lacking to determine whether postponing or substituting procedures could be safely generalized beyond the crisis context. Additionally, it remains unclear to what extent primary care providers, such as general practitioners, were able to absorb deferred patient demand or whether substantial unmet needs persisted. Further investigation into these dynamics is warranted, as they may inform the evolution of care pathways and guideline development in post-pandemic health systems [1].

Notable changes included increased use of telephone consultations, which may have allowed continued clinical engagement despite reduced physical access. It is in line with earlier published articles about the increased use of telehealth [39–41]. This suggests that outpatient visits and follow-up adapted rapidly to pandemic constraints and could provide a model for reducing unnecessary physical visits in the future.

We investigated whether treatment mix changes were geographically patterned, reflecting the variable impact of COVID-19 across Dutch regions. Regional differences in infection rates and ICU occupancy during the pandemic have been well documented [42–44]. It is likely that hospitals in regions with higher COVID-19 burdens were forced to defer or redirect more elective care, such as benign gynecological care. For instance, the southern provinces, including Noord-Brabant and Limburg, experienced high ICU pressure during early pandemic waves, which may have prompted more aggressive triage and substitution compared to less affected regions. However, we did not observe substantial regional variation in treatment mix. This may reflect the uniformly applied deferral policy and the equal distribution of COVID-19 hospitalized patients nationwide [43–45].

We also assessed the hospital type – academic medical centers, top-clinical hospitals, general hospitals and ITCs – on the extent of care modification. Academic hospitals played a central role in pandemic response. They were likely to reallocate operating rooms, anesthesia personnel, and ward capacity to COVID-related care, thereby reducing availability for elective gynecological procedures [4,46]. In contrast, ITCs, which typically do not have emergency or ICU capacity and are focused on elective procedures, may have been less disrupted. Indeed, Dutch health authorities encouraged the use of ITCs during later phases of the pandemic to manage backlogs and restore access to deferred care [39,47,48]. Our results show that care delivery remained stable across all four care pathways. Only a minor increase for ITCs was seen over the years, taking over from academic hospitals, with no significant increase in the initial COVID-19 phase. This suggests that ITCs—despite being less involved in acute COVID care—were not consistently leveraged to absorb overflow [4]. This finding echoes concerns that system-level integration of ITCs remains suboptimal, particularly during periods of constrained capacity [49].

Taken together, our findings suggest that the Dutch health system prioritized continuity within pathways rather than radical reorganization. Efforts to minimize harm focused on preserving existing access where possible, rather than shifting care between settings or modalities. While this conservative approach may have protected core services, it also highlights missed opportunities to trial alternative, potentially more sustainable forms of care under real-world constraints.

Although our analyses focused on benign gynaecologic care, the underlying mechanisms are likely not unique to this specialty. The care we studied largely concerned planned, non‑urgent procedures and consultations, where alternative treatment options, potential substitution or shifting care towards primary care are already part of routine decision‑making. It is therefore plausible that similar patterns of limited restructuring and an emphasis on continuity rather than transformation would be observed in other forms of elective, non‑life‑threatening care in which shared decision‑making about treatment options is common. At the same time, generalizability to settings involving life‑threatening conditions, severe morbidity, or the absence of evidence‑based alternatives in primary care may be limited, as perceived urgency, acceptable risk, and ethical considerations differ fundamentally in those contexts. Future research should examine whether and how care delivery adaptations during crises differ between elective and urgent care pathways, and across other medical disciplines.

This study adds to ongoing discussions about the resilience of elective care and its readiness to adapt under pressure [19,20]. Rather than large-scale innovation or substitution, we observed small, pragmatic adjustments, such as increased teleconsultations and limited decentralization. These experiences provide insight into the strengths and constraints of current care models and offer valuable lessons for improving flexibility and preparedness [50].

### Strengths and limitations

This study’s major strength lies in its nationwide scope and the longitudinal comparison across six years. By integrating DHD and Vektis data, we capture real-world treatment patterns across diverse hospital types and regions. Furthermore, the DHD source adheres to uniform data registration procedures, which strengthens the consistency and comparability of our results. Another strength lies in its ability to detect system-level signals of adaptation aimed at minimizing patient harm, by analyzing trends in care delivery rather than just volume changes. Additionally, by analyzing a diverse set of gynecological care pathways, from menstrual disorders to pelvic organ prolapse, this study provides nuanced insights into how various clinical contexts were impacted during the pandemic.

Despite its strengths, this study has several important limitations. As an observational analysis, it cannot establish causal relationships between the pandemic and observed shifts in care patterns. Moreover, we lacked information on clinical severity, which constrains our ability to determine whether hospitals triaged patients based on urgency or complexity. We also did not capture potential shifts to primary care, which may have absorbed part of the deferred demand, nor did we assess patient outcomes or satisfaction. These limitations hinder our ability to evaluate the appropriateness or long-term impact of reduced or altered treatment, particularly in cases where care was delayed or forgone.

### Implications for practice

The COVID-19 pandemic placed considerable pressure on the organizational capacity of benign gynecological care in the Netherlands. Our findings suggest that, despite limited resources, the healthcare system largely preserved access for comparable patient populations through targeted and modest adjustments in care delivery, such as increased use of telephone consultations. However, the absence of broader adaptation, such as proactive triage, strategic substitution or greater use of ITCs, highlights the need to strengthen flexibility in care organization during future crisis. Strengthening the ability to redirect patients or modalities in real-time could help mitigate harm more effectively.

One critical question that remains is whether the observed decline in hospital-based treatments reflects forgone care or a redistribution of services towards primary care. General practitioners may have played a compensatory role by managing symptoms, postponing referrals, or offering conservative management options such as medication or watchful waiting. Yet, national-level data on general practice activity and referral patterns during the pandemic are lacking. Future research should therefore investigate trends in primary care to determine how demand was managed outside of the hospital setting.

In parallel, qualitative research is essential to understand how patients experienced care constraints and whether they perceived delays or adjustments as acceptable or harmful. Did they feel adequately supported in the absence of standard treatment? Did conservative management meet their expectations? How did they experience the shift toward telehealth care? Such insights are crucial to inform future redesign of benign gynecological care pathways that are both resilient and responsive to patient needs in times of limited capacity.

## Conclusion

While the COVID-19 pandemic severely restricted surgical capacity for benign gynecological care in the Netherlands, we observed little in the way of strategic triage to select patients who stood to benefit most from the remaining capacity. Our findings suggest that the Dutch healthcare system focused on preserving continuity rather than restructuring care delivery. Targeted adaptations, such as the use of telephone consultations and outpatient alternatives, may have helped minimize patient harm, but broader system flexibility was limited. As future shocks and ongoing capacity constraints loom, building a more responsive care model will be essential to protect access to and quality of care for all patient groups.

## Supporting information

S1 File Treatment mix in proportion to the total of these selected activities per region per year. Definition of regions (in provinces): North = Drenthe, Friesland, Groningen. Middle = Flevoland, Gelderland, Overijssel, Noord-Holland, Utrecht, Zuid-Holland, Noord-Holland. South = Maastricht, Noord-Brabant, Zeeland.
(DOCX)
