# Supplementary material for: Evaluating patient harm minimization during the COVID-19-driven reduction in benign gynecological care: a nationwide claims-based longitudinal study in the Netherlands
Source: PLoS One. 2026 Apr 1;21(4):e0345619. doi: 10.1371/journal.pone.0345619 (PMC13042642; doi:10.1371/journal.pone.0345619)
Supplement: S1 File — Definition of regions (in provinces): North = Drenthe, Friesland, Groningen. Middle = Flevoland, Gelderland, Overijssel, Noord-Holland, Utrecht, Zuid-Holland, Noord-Holland. South = Maastricht, Noord-Brabant, Zeeland. (DOCX) [file pone.0345619.s001.docx]

**S1 File. Treatment mix in proportion to the total of these selected activities per region per year (2017 – 2022).**

Definition of regions (in provinces):

North = Drenthe, Friesland, Groningen

Middle = Flevoland, Gelderland, Overijssel, Noord-Holland, Utrecht, Zuid-Holland, Noord-Holland

South = Maastricht, Noord-Brabant, Zeeland

**Table A. Heavy menstrual bleeding.**

|  | | **2017** | **2018** | **2019** | **2020** | **2021** | **2022** |
| --- | --- | --- | --- | --- | --- | --- | --- |
| North |  |  |  |  |  |  |  |
|  | Implanon insertion | <0.20 | <0.20 | 0.27 | 0.20 | 0.21 | 0.54 |
|  | Intrauterine device insertion | 7.47 | 7.23 | 7.37 | 8.40 | 8.26 | 8.89 |
|  | diagnostic hysteroscopy | 2.28 | 2.66 | 4.02 | 3.20 | 2.27 | 2.70 |
|  | therapeutic hysteroscopy | 21.04 | 19.36 | 17.56 | 27.00 | 25.21 | 25.07 |
|  | hysteroscopic myomectomy (TCRM) type a | 15.03 | 13.40 | 12.33 | 10.00 | 8.88 | 11.05 |
|  | hysteroscopic myomectomy (TCRM) type b | 35.25 | 39.15 | 40.35 | 34.40 | 39.26 | 42.32 |
|  | diagnostic laparoscopy | 1.37 | 1.49 | 2.55 | 1.00 | 2.89 | 2.16 |
|  | total laparoscopic hysterectomy | 12.66 | 12.45 | 11.53 | 11.80 | 9.30 | 4.85 |
|  | vaginal hysterectomy | 3.37 | 3.40 | 2.82 | 3.00 | 3.31 | 1.35 |
|  | abdominal hysterectomy | 1.55 | 0.85 | 1.21 | 1.00 | 0.41 | 1.08 |
| Middle |  |  |  |  |  |  |  |
|  | Implanon insertion | 0.32 | 0.65 | 0.43 | 0.40 | 0.40 | 0.63 |
|  | Intrauterine device insertion | 7.55 | 8.48 | 9.69 | 9.41 | 11.24 | 9.90 |
|  | diagnostic hysteroscopy | 4.92 | 4.36 | 4.58 | 5.43 | 4.48 | 4.45 |
|  | therapeutic hysteroscopy | 22.45 | 21.74 | 23.93 | 23.74 | 22.83 | 22.24 |
|  | hysteroscopic myomectomy (TCRM) type a | 13.13 | 12.33 | 12.99 | 13.86 | 16.41 | 17.58 |
|  | hysteroscopic myomectomy (TCRM) type b | 33.48 | 35.56 | 34.07 | 33.02 | 32.69 | 34.66 |
|  | diagnostic laparoscopy | 1.51 | 1.51 | 1.42 | 1.48 | 1.58 | 1.80 |
|  | total laparoscopic hysterectomy | 10.49 | 10.20 | 9.15 | 9.21 | 7.94 | 6.21 |
|  | vaginal hysterectomy | 4.76 | 3.90 | 2.43 | 2.56 | 1.88 | 1.97 |
|  | abdominal hysterectomy | 1.38 | 1.27 | 1.33 | 0.88 | 0.56 | 0.55 |
| South |  |  |  |  |  |  |  |
|  | Implanon insertion | 0.49 | 0.84 | 0.74 | <0.20 | 0.41 | 0.82 |
|  | Intrauterine device insertion | 7.52 | 6.54 | 10.17 | 10.41 | 10.40 | 11.93 |
|  | diagnostic hysteroscopy | 3.84 | 3.18 | 3.57 | 4.71 | 2.88 | 3.57 |
|  | therapeutic hysteroscopy | 19.48 | 18.07 | 16.97 | 17.31 | 17.10 | 14.54 |
|  | hysteroscopic myomectomy (TCRM) type a | 11.58 | 12.24 | 11.65 | 12.71 | 14.52 | 14.68 |
|  | hysteroscopic myomectomy (TCRM) type b | 33.97 | 37.64 | 39.60 | 37.46 | 39.03 | 39.37 |
|  | diagnostic laparoscopy | 1.21 | 1.56 | 1.35 | 1.75 | 1.65 | 2.47 |
|  | total laparoscopic hysterectomy | 12.79 | 12.91 | 11.25 | 10.84 | 10.30 | 9.47 |
|  | vaginal hysterectomy | 7.85 | 5.94 | 4.24 | 3.83 | 3.09 | 2.33 |
|  | abdominal hysterectomy | 1.26 | 1.08 | 0.47 | 0.99 | 0.62 | 0.82 |

**Table B. Uterine fibroids.**

|  | | **2017** | **2018** | **2019** | **2020** | **2021** | **2022** |
| --- | --- | --- | --- | --- | --- | --- | --- |
| North |  |  |  |  |  |  |  |
|  | Intrauterine device insertion | 0.94 | 1.61 | <0.20 | 1.10 | 1.00 | 3.23 |
|  | diagnostic hysteroscopy | 1.42 | 0.81 | 0.70 | 5.49 | 2.00 | 1.61 |
|  | therapeutic hysteroscopy | 7.08 | 8.87 | 9.15 | 9.89 | 11.00 | 12.90 |
|  | uterine artery embolization | 0.47 | <0.20 | <0.20 | <0.20 | <0.20 | <0.20 |
|  | hysteroscopic myomectomy (TCRM) type a | 15.57 | 8.06 | 7.04 | 7.69 | 6.00 | 17.74 |
|  | hysteroscopic myomectomy (TCRM) type b | 14.62 | 17.74 | 31.69 | 18.68 | 31.00 | 27.42 |
|  | myomectomy | 3.30 | 4.84 | 4.23 | 7.69 | 3.00 | <0.20 |
|  | diagnostic laparoscopy | 0.47 | <0.20 | <0.20 | 1.10 | <0.20 | <0.20 |
|  | total laparoscopic hysterectomy | 24.06 | 23.39 | 23.24 | 21.98 | 23.00 | 14.52 |
|  | vaginal hysterectomy | 2.36 | <0.20 | 2.11 | 1.10 | 1.00 | 1.61 |
|  | abdominal hysterectomy | 29.72 | 34.68 | 21.83 | 25.27 | 22.00 | 20.97 |
| Middle |  |  |  |  |  |  |  |
|  | Intrauterine device insertion | 1.72 | 1.96 | 1.25 | 2.21 | 1.99 | 1.55 |
|  | diagnostic hysteroscopy | 3.04 | 3.10 | 2.87 | 2.52 | 3.14 | 2.40 |
|  | therapeutic hysteroscopy | 8.36 | 8.10 | 10.66 | 11.67 | 11.22 | 9.32 |
|  | uterine artery embolization | 0.35 | 0.32 | 0.44 | 0.42 | 0.21 | 0.28 |
|  | hysteroscopic myomectomy (TCRM) type a | 12.47 | 9.94 | 11.84 | 12.20 | 14.78 | 14.97 |
|  | hysteroscopic myomectomy (TCRM) type b | 22.50 | 23.10 | 23.82 | 25.13 | 24.21 | 26.98 |
|  | myomectomy | 8.51 | 8.54 | 6.25 | 6.31 | 8.60 | 8.33 |
|  | diagnostic laparoscopy | 0.76 | 1.20 | 0.81 | 0.84 | 0.84 | 0.85 |
|  | total laparoscopic hysterectomy | 21.49 | 21.77 | 20.59 | 19.77 | 16.25 | 15.68 |
|  | vaginal hysterectomy | 2.43 | 1.27 | 1.84 | 1.05 | 1.05 | 1.13 |
|  | abdominal hysterectomy | 18.35 | 20.70 | 19.63 | 17.88 | 17.71 | 18.50 |
| South |  |  |  |  |  |  |  |
|  | Intrauterine device insertion | 2.29 | 1.15 | 2.02 | 2.64 | 2.01 | 3.43 |
|  | diagnostic hysteroscopy | 3.89 | 1.84 | 3.46 | 2.64 | 1.20 | 3.00 |
|  | therapeutic hysteroscopy | 7.55 | 8.99 | 6.05 | 10.57 | 6.43 | 6.44 |
|  | uterine artery embolization | 0.23 | 0.23 | 0.58 | 0.75 | 0.40 | <0.20 |
|  | hysteroscopic myomectomy (TCRM) type a | 12.81 | 16.82 | 13.26 | 12.45 | 15.66 | 22.75 |
|  | hysteroscopic myomectomy (TCRM) type b | 17.39 | 17.74 | 21.61 | 18.11 | 27.71 | 23.61 |
|  | myomectomy | 5.03 | 5.53 | 5.19 | 4.91 | 6.02 | 4.72 |
|  | diagnostic laparoscopy | 1.83 | 4.15 | 2.59 | 0.75 | 2.81 | 0.86 |
|  | total laparoscopic hysterectomy | 24.71 | 23.27 | 21.61 | 24.53 | 16.87 | 12.88 |
|  | vaginal hysterectomy | 2.52 | 2.07 | 3.17 | 2.26 | 2.01 | 1.29 |
|  | abdominal hysterectomy | 21.74 | 18.20 | 20.46 | 20.38 | 18.88 | 21.03 |

**Table C. Pelvic organ prolapse**

|  | | **2017** | **2018** | **2019** | **2020** | **2021** | **2022** |
| --- | --- | --- | --- | --- | --- | --- | --- |
| North |  |  |  |  |  |  |  |
|  | total laparoscopic hysterectomy | 0.23 | <0.2 | 0.64 | 0.50 | <0.2 | <0.2 |
|  | vaginal hysterectomy | 1.71 | 2.25 | 1.76 | 0.25 | 0.27 | 0.61 |
|  | a&p colporrhaphy | 38.38 | 36.90 | 38.46 | 41.25 | 40.16 | 38.30 |
|  | a&p colporrhaphy with cervical amputation | 7.06 | 8.17 | 13.46 | 11.25 | 18.03 | 17.33 |
|  | a&p colporrhaphy with vaginal hysterectomy | 14.69 | 13.24 | 10.10 | 10.00 | 8.47 | 3.95 |
|  | a/p colporrhaphy with mesh placement | 0.57 | <0.2 | <0.2 | 0.25 | <0.2 | <0.2 |
|  | a/p colporrhaphy with double mesh placement | <0.2 | <0.2 | <0.2 | <0.2 | <0.2 | 0.61 |
|  | tvt/tot placement | 14.24 | 12.82 | 13.62 | 12.75 | 11.20 | 13.98 |
|  | tvt/tot + a/p colporrhaphy | 1.37 | 1.13 | <0.2 | 0.50 | 0.55 | 1.52 |
|  | enterocele repair abd/vag | 0.91 | 3.10 | 2.24 | 2.00 | 1.09 | 1.52 |
|  | vaginal vault suspension | 1.25 | 0.99 | 0.32 | 0.75 | 0.27 | <0.2 |
|  | sacrocolpopexy | 1.03 | 1.55 | 2.56 | 2.00 | 2.19 | 1.82 |
|  | sacrocolpopexy + a/p colporrhaphy | 15.60 | 17.46 | 13.62 | 15.75 | 15.03 | 20.06 |
|  | sacrocolpopexy + a&p colporrhaphy | 1.82 | 1.41 | 0.80 | 1.00 | 1.64 | <0.2 |
|  | laparoscopic sacrocolpopexy | 1.03 | 0.85 | 2.08 | 1.75 | 1.09 | 0.30 |
| Middle |  |  |  |  |  |  |  |
|  | total laparoscopic hysterectomy | 0.77 | 0.70 | 0.58 | 0.70 | 0.49 | 0.36 |
|  | vaginal hysterectomy | 1.67 | 1.42 | 1.27 | 1.60 | 1.70 | 1.14 |
|  | a&p colporrhaphy | 32.36 | 31.61 | 30.54 | 35.51 | 33.59 | 35.60 |
|  | a&p colporrhaphy with cervical amputation | 6.63 | 7.04 | 7.21 | 7.02 | 7.68 | 8.62 |
|  | a&p colporrhaphy with vaginal hysterectomy | 9.50 | 10.64 | 8.34 | 6.57 | 6.08 | 5.73 |
|  | a/p colporrhaphy with mesh placement | 1.47 | 1.25 | 0.77 | 0.55 | 0.44 | 0.46 |
|  | a/p colporrhaphy with double mesh placement | 0.90 | 0.52 | 0.22 | <0.2 | 0.34 | 0.31 |
|  | tvt/tot placement | 16.29 | 17.63 | 17.79 | 16.40 | 13.66 | 13.67 |
|  | tvt/tot + a/p colporrhaphy | 1.67 | 1.42 | 1.44 | 0.70 | 1.17 | 1.08 |
|  | enterocele repair abd/vag | 0.65 | 0.42 | 0.72 | 0.55 | 0.88 | 0.93 |
|  | vaginal vault suspension | 0.47 | 0.25 | 0.22 | <0.2 | <0.2 | <0.2 |
|  | sacrocolpopexy | 1.94 | 1.57 | 2.32 | 2.16 | 1.94 | 1.65 |
|  | sacrocolpopexy + a/p colporrhaphy | 21.62 | 22.12 | 24.36 | 24.47 | 28.73 | 28.22 |
|  | sacrocolpopexy + a&p colporrhaphy | 1.06 | 0.95 | 1.38 | 0.75 | 1.12 | 0.36 |
|  | laparoscopic sacrocolpopexy | 1.94 | 1.57 | 2.32 | 2.16 | 1.94 | 1.65 |
| South |  |  |  |  |  |  |  |
|  | total laparoscopic hysterectomy | 0.20 | 0.23 | <0.2 | <0.2 | <0.2 | 0.33 |
|  | vaginal hysterectomy | 2.17 | 1.86 | 0.99 | 2.42 | 1.22 | 0.33 |
|  | a&p colporrhaphy | 30.81 | 29.74 | 29.41 | 28.90 | 33.69 | 29.04 |
|  | a&p colporrhaphy with cervical amputation | 6.31 | 4.96 | 6.55 | 6.85 | 8.99 | 10.23 |
|  | a&p colporrhaphy with vaginal hysterectomy | 12.48 | 12.70 | 8.95 | 9.01 | 8.69 | 6.27 |
|  | a/p colporrhaphy with mesh placement | 0.66 | 0.46 | 0.50 | 1.88 | 0.61 | 1.82 |
|  | a/p colporrhaphy with double mesh placement | <0.2 | <0.2 | 0.33 | <0.2 | <0.2 | 0.33 |
|  | tvt/tot placement | 18.20 | 18.28 | 17.48 | 16.53 | 17.23 | 18.65 |
|  | tvt/tot + a/p colporrhaphy | 1.71 | 2.71 | 1.57 | 1.21 | 0.61 | 1.32 |
|  | enterocele repair abd/vag | 1.05 | 0.46 | 1.08 | 0.94 | 0.91 | 0.99 |
|  | vaginal vault suspension | 0.85 | 1.08 | 0.33 | 1.08 | 0.61 | 1.16 |
|  | sacrocolpopexy | 1.25 | 1.70 | 1.74 | 1.34 | 1.52 | 1.32 |
|  | sacrocolpopexy + a/p colporrhaphy | 21.42 | 23.08 | 28.25 | 26.88 | 23.78 | 26.90 |
|  | sacrocolpopexy + a&p colporrhaphy | 0.85 | 0.46 | 0.66 | 0.40 | 0.91 | <0.2 |
|  | laparoscopic sacrocolpopexy | 1.91 | 2.09 | 2.07 | 2.28 | 1.07 | 1.32 |

**Table D. First trimester pregnancy complications**

|  | | **2017** | **2018** | **2019** | **2020** | **2021** | **2022** |
| --- | --- | --- | --- | --- | --- | --- | --- |
| North |  |  |  |  |  |  |  |
|  | hysteroscopy | 1.29 | 3.76 | 5.58 | 10.10 | 13.08 | 13.23 |
|  | uterine curettage | 9.41 | 7.52 | 7.52 | 7.17 | 13.85 | 10.05 |
|  | surgical abortion/evacuation of retrained products of conception | 74.91 | 72.86 | 72.82 | 67.75 | 53.85 | 57.67 |
|  | laparoscopic surgery ectopic pregnancy | 13.84 | 15.03 | 12.62 | 13.68 | 17.31 | 16.93 |
|  | open surgery ectopic pregnancy | 0.55 | 0.84 | 1.46 | 1.30 | 1.92 | 2.12 |
| Middle |  |  |  |  |  |  |  |
|  | hysteroscopy | 1.98 | 2.52 | 6.83 | 9.58 | 11.57 | 10.67 |
|  | uterine curettage | 9.31 | 8.66 | 7.96 | 6.94 | 9.87 | 11.07 |
|  | surgical abortion/evacuation of retrained products of conception | 73.57 | 73.73 | 67.64 | 63.38 | 58.35 | 55.30 |
|  | laparoscopic surgery ectopic pregnancy | 14.50 | 14.18 | 16.83 | 19.12 | 19.23 | 22.21 |
|  | open surgery ectopic pregnancy | 0.65 | 0.91 | 0.73 | 0.97 | 0.98 | 0.75 |
| South |  |  |  |  |  |  |  |
|  | hysteroscopy | 2.41 | 3.91 | 8.36 | 9.021 | 15.57 | 13.32 |
|  | uterine curettage | 9.94 | 10.69 | 8.64 | 8.45 | 9.38 | 13.04 |
|  | surgical abortion/evacuation of retrained products of conception | 70.19 | 68.45 | 63.09 | 60.65 | 51.60 | 49.18 |
|  | laparoscopic surgery ectopic pregnancy | 16.00 | 16.82 | 18.94 | 21.11 | 23.03 | 24.46 |
|  | open surgery ectopic pregnancy | 1.46 | <0.2 | 0.97 | 0.77 | 0.43 | <0.2 |
